# Pulmonary injury risk curves and behavioral changes from blast overpressure exposures of varying frequency and intensity in rats

**DOI:** 10.1038/s41598-020-73643-7

**Published:** 2020-10-06

**Authors:** Venkatasivasai Sujith Sajja, Jonathan K. Statz, LCDR Peter B. Walker, Irene D. Gist, Donna M. Wilder, Stephen T. Ahlers, Joseph B. Long

**Affiliations:** 1 Blast Induced Neurotrauma Branch, Center for Military Psychiatry and Neurosciences, Walter Reed Army Institute of Research, Silver Spring, MD USA; 2 Neurotrauma Department, Naval Medical Research Center, Silver Spring, MD USA; 3 The Henry M. Jackson Foundation for the Advancement of Military Medicine, Inc., Bethesda, MD USA; 4 The Geneva Foundation, Tacoma, WA USA

**Keywords:** Biomedical engineering, Trauma

## Abstract

At present, there are no set guidelines establishing cumulative limits for blast exposure numbers and intensities in military personnel, in combat or training operations. The objective of the current study was to define lung injury, pathology, and associated behavioral changes from primary repeated blast lung injury under appropriate exposure conditions and combinations (i.e. blast overpressure (BOP) intensity and exposure frequency) using an advanced blast simulator. Male Sprague Dawley rats were exposed to BOP frontally and laterally at a pressure range of ~ 8.5–19 psi, for up to 30 daily exposures. The extent of lung injury was identified at 24 h following BOP by assessing the extent of surface hemorrhage/contusion, Hematoxylin and Eosin staining, and behavioral deficits with open field activity. Lung injury was mathematically modeled to define the military standard 1% lung injury threshold. Significant levels of histiocytosis and inflammation were observed in pressures ≥ 10 psi and orientation effects were observed at pressures ≥ 13 psi. Experimental data demonstrated ~ 8.5 psi is the threshold for hemorrhage/contusion, up to 30 exposures. Modeling the data predicted injury risk up to 50 exposures with intensity thresholds at 8 psi for front exposure and 6psi for side exposures, which needs to be validated further.

## Introduction

Exposure to blast from improvised explosive devices (IEDs) has been recognized as the major cause of injuries to systemic organs and brain, and is associated with numerous disabilities in the recent wars in Iraq and Afghanistan^1^. Blast exposures to military personnel are not restricted to IED explosions in wartime, but can also result from blast overpressure (BOP) exposures from various weapon systems and breaching of structures in training. “Safe levels” of BOP are estimated based upon thresholds for minor tympanic membrane rupture (small tears and/or holes) for an unprotected ear at 3 psi^2^. A recent research report has shown that training in breaching and heavy weapon system operations can lead to BOP exposures that exceed 13 psi static levels^3^. Moreover, the blast exposure experienced in operational breaching, in the operation of heavy weapon systems in the threat environment, and by explosive ordinance personnel (EOD) in theater could far exceed 13 psi static levels due to the close proximity to these blasts compared to what is experienced in training. In addition, there is mounting concern that repeated exposure to even low-intensity BOP (overpressure that does not result in overt damage to organs other than ears) across a wide variety of weapon systems in a military career can result in cumulative, long-term neurological effects^3–5^.

It is critical to develop empirical risk assessment guidelines based upon quantitative parameters, such as gauge measurements, that permit the formulation of cumulative exposure–response relationships. At present there are no set guidelines establishing cumulative limits for repetitive BOP exposures for military personnel in combat or training operations based upon potentially deleterious effects on the brain beyond the acute exposure standards currently in use. Moreover, the cumulative effects of BOP on other blast-sensitive organs such as the lung are also undefined, despite the likelihood that the lung may be especially susceptible to disruption with repeated exposures. A recent epidemiological study has shown a stronger association between BOP exposure and cardio-respiratory symptoms than the burn-pit smoke exposure^6^. It is important to understand BOP exposure conditions in which blast-sensitive organs, notably the lung, are compromised or injured. Although lung injury and associated pathophysiology in rats has been characterized across a range of BOP intensities generated in constant diameter shock tubes^7–9^, which produce blast pressure profiles that bear little resemblance to the conditions experienced by Warfighter^10,11^, the potential cumulative effects of repeated BOP on the lung and the consequent pathological and behavioral changes have not been investigated comprehensively.

The objective of the current study was to define lung injury, pathology, and associated behavioral changes from the primary repeated blast lung injury under appropriate exposure conditions and combinations (i.e. BOP intensity and exposure frequency). This data has been used to develop risk curves of cumulative effects of primary BOP exposure where BOP-induced trauma and brain perturbations may not be readily apparent upon initial evaluation/diagnosis, which is primarily relevant to Warfighters in operational and training settings. A well-characterized experimental rat model of BOP injury was used to characterize the cumulative disruptive effects of BOP exposure-scaled injury thresholds using an advanced blast simulator (ABS), which creates flow conditions very closely resembling the Friedlander waves produced in the “free field” by improvised explosive devices (IEDs) and other explosive detonations. This represents a first crucial step toward defining occupational guidelines and standards for Warfighters exposed to BOP. In addition, the role of orientation (frontal vs side-on) on systemic cumulative effects of BOP exposure were assessed. The data were mathematically modeled to empirically define lung injury risk curves using a rodent model of blast.

## Results

### Open field assessment

**Figure 1:**
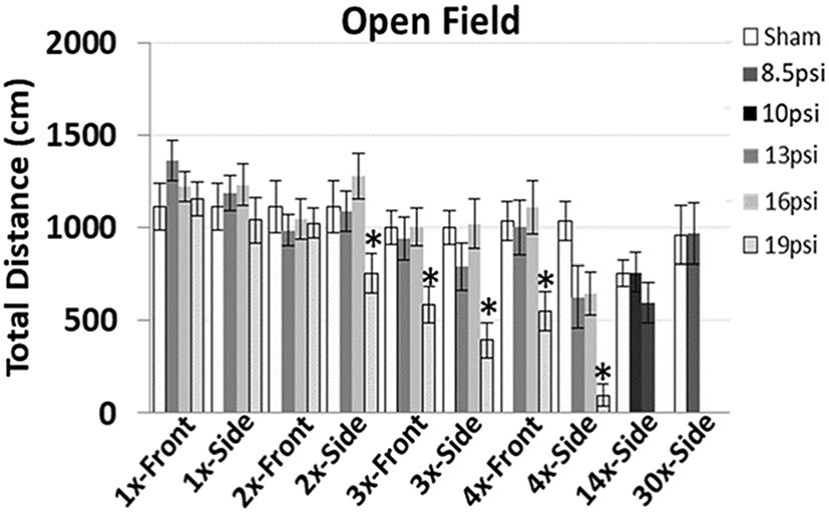
A significant decrease in the open field activity was observed only with repeated 19 psi exposures (2 ×-side, 3 ×–4 × front and side exposures), but not with single exposures at any pressure. In addition, no changes were observed following repeated BOP exposures ranging from 8.5–16psi.

At 24 h following BOP exposure, 2 ×–4 × side- and 3 ×–4 × frontal-19 psi exposure groups had significant reductions in activity. In contrast, 14 ×–8.5 psi, 14 ×–10 psi, and 30 ×–8.5 psi side exposure groups did not show any change in activity at 24 h following final BOP exposures (p < 0.05, Fig. 1).

### Lung injury assessments

**Figure 2:**
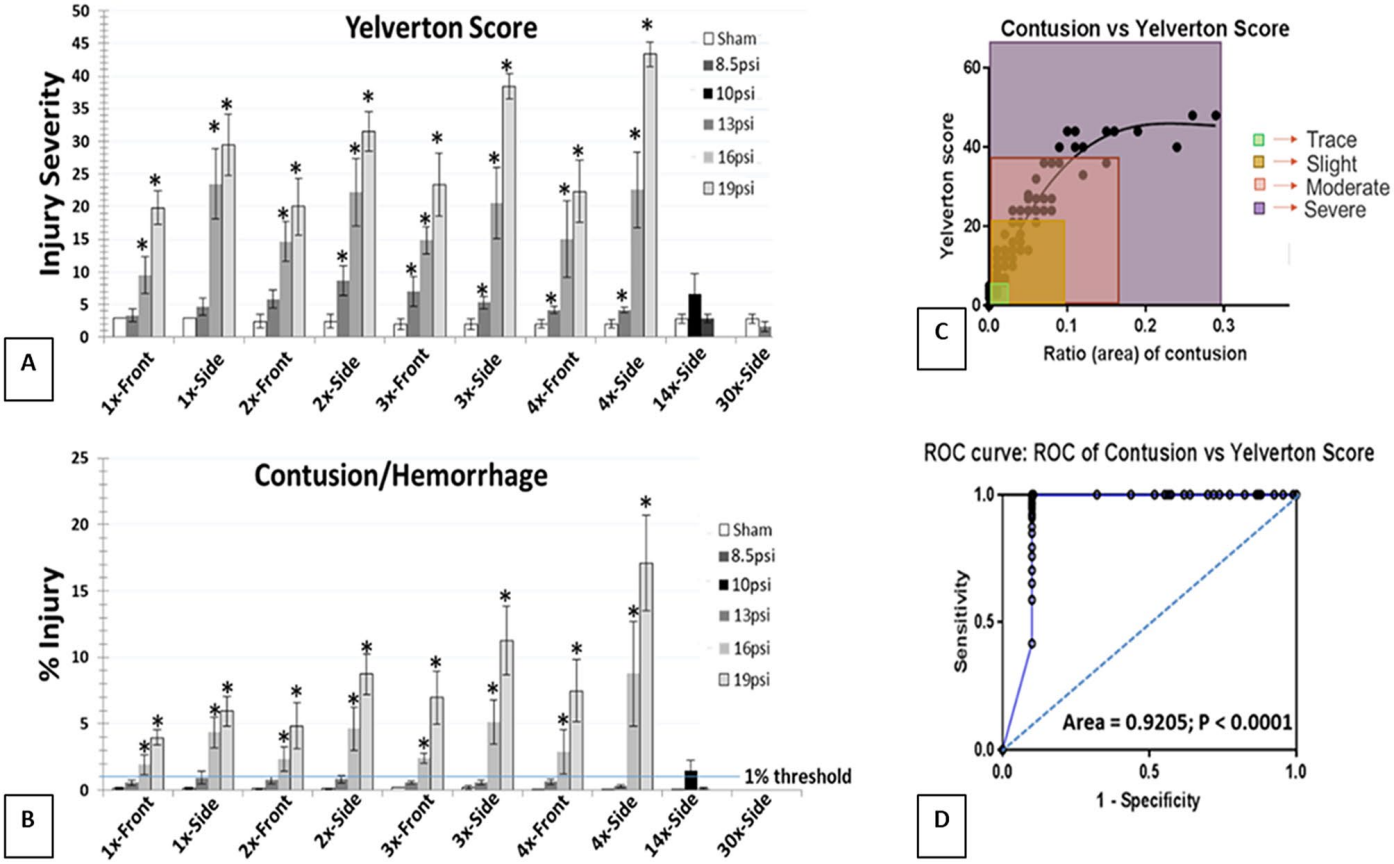
Cumulative effects of repeated BOP. (**A**,**B**) Gradient raise in the lung injury levels with increase in frequency of BOP, and orientation of the animal to the BOP exposure has an effect on the extent of injury. (**C**,**D**) Significant correlation between Yelverton score and MATLAB-scoring shows that surface lung injury is a significant predictor of overall lung injury.

Using MATLAB 2015b-based image characterizations for gross macroscopic assessments, significant increases in the percentage area of lung injury (as reflected by contusion/hemorrhage) were evident in the 13, 16, and 19 psi frontal and side exposure groups that were subjected to 1 ×–4 × daily exposures (Fig. 2A, p < 0.05). Lung injuries sustained by the side exposure groups generally trended higher with relatively higher pressure (> 13 psi); however, only the 4×–19psi groups revealed a significant difference between front and side exposures. This orientation and cumulative effect on lung injury was not observed at 13 psi up to 4 × exposures but exceeded a mean % injury that exceeded our defined 1% injury threshold, a standard that is used in Warfighter health hazard assessment. Similarly, the group of rats exposed to 14x-10 psi exposures had exceeded 1% injury threshold, the measurements were not significantly different from those made in sham injured rats. Similarly, no indications of lung injury were observed in the 14x- or 30x-8.5psi treatment groups. Rats that were exposed to shock waves in a side orientation had significantly greater injuries than were observed in rats exposed to comparable magnitudes and frequencies of the exposures in a frontal orientation, demonstrating a pronounced orientation effect. Analyses of Yelverton scores showed similar patterns of injury outcomes as were observed with MATLAB 2015b image processing (Fig. 2B). In addition, percentage injury data from MATLAB 2015b image processing (semi-quantitative process) had significant correlation with Yelverton scores, which are derived from subjective scoring (R^2^ = 0.944, p < 0.0001) (Fig. 2C,D).

### Pathological assessments from H&E staining

**Figure 3:**
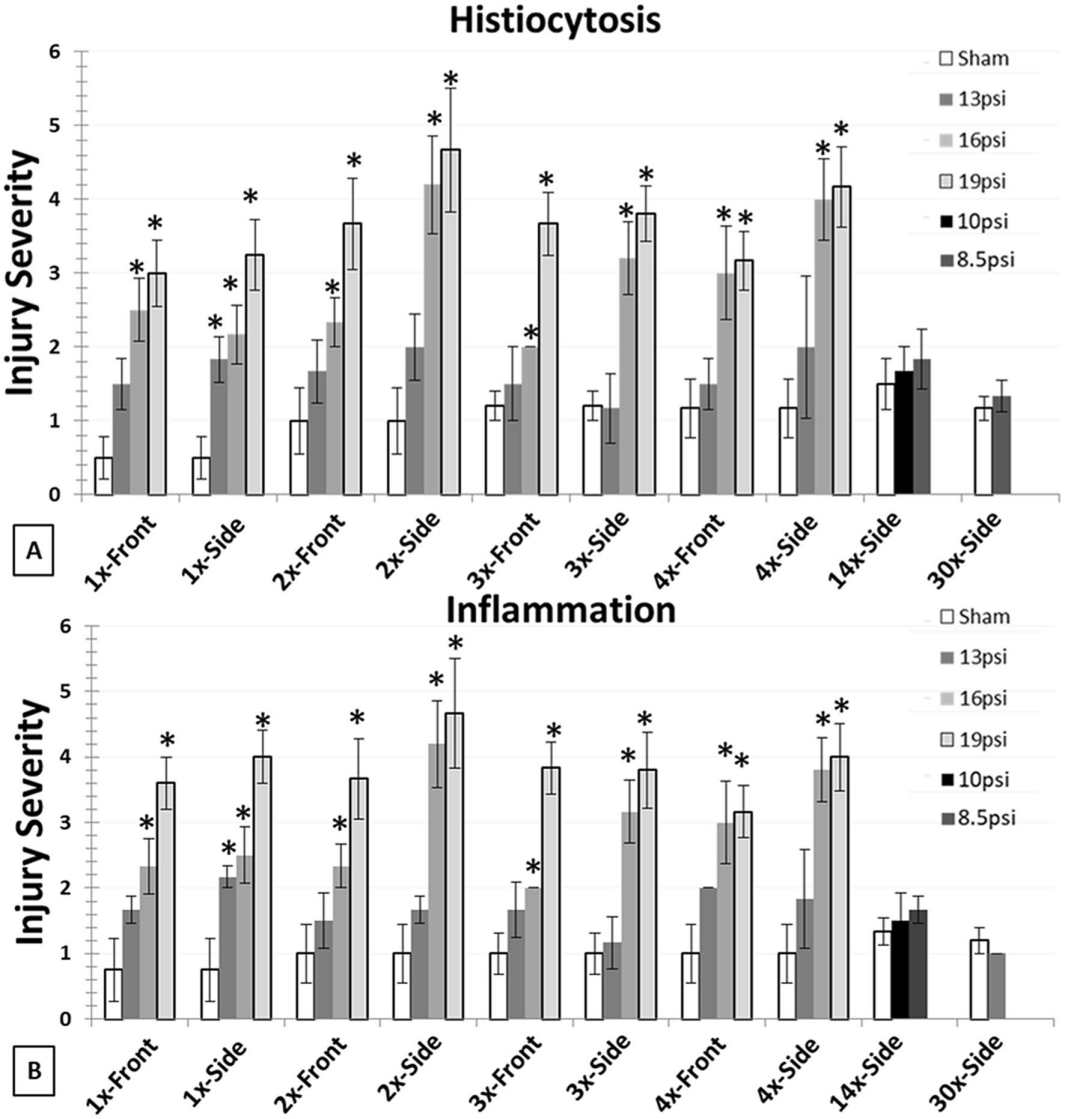
Significant increases in lung histiocytosis and inflammation were observed at 1 ×–4 × 16 and 19 psi pressure groups; however, inflammation and histiocytosis was observed to be increased in 1 ×–13 side group but no changes were observed in other groups of 13 psi pressure groups, 14 × (8.5 and 10 psi) and 30 × (8.5 psi) pressure group.

A significant increase in lung inflammation and histiocytosis was observed in 1 ×–4 × front and side exposures of 16 and 19psi. A significant increase in inflammation and histiocytosis was observed in 1 ×–13 psi side group, but not 1 ×–13 psi front group (p < 0.05, Fig. 3). No changes were observed in the 14 × and 30 × exposure groups when compared to sham.

### Mathematical modelling

**Figure 4:**
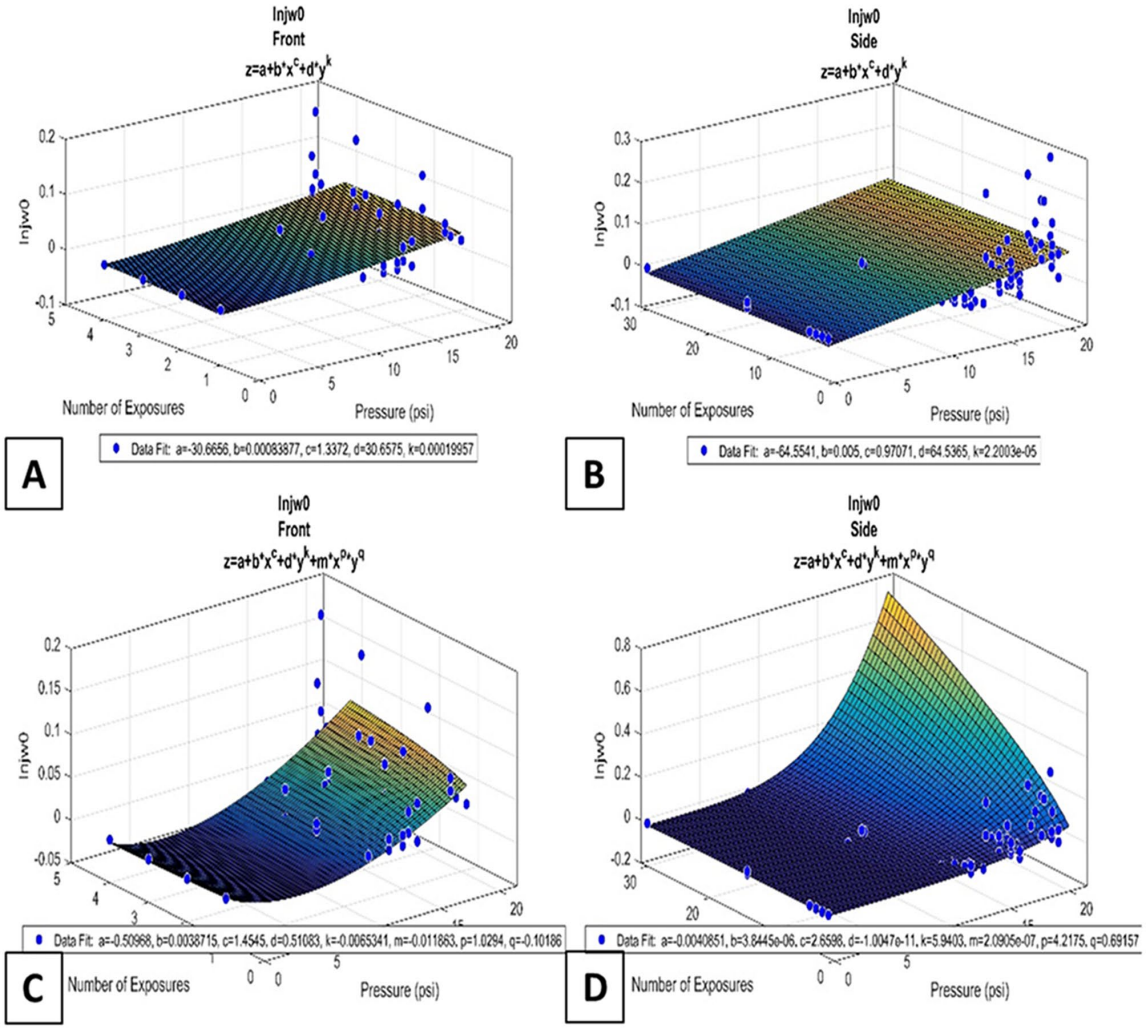
3D power function models of percent lung injury as a function of number of exposures and pressure of those exposures. Equations and values for coefficients are in the corresponding plot. (**A**,**B**) No interaction term models; (**C**,**D**) with interaction term models; (**A**,**C**) front orientation models; Right: side orientation models.

The R^2^ values for the 2D models are in Table 1 below, while the area under the curves of the ROC curves for the 3D models (of which the power function versions are shown in Fig. 4) of the lung injury models are in Table 2. In Table 1, the cells with N/A indicate that the data for that model and/or variable were constant for all subject data points in that group, and thus a model could not be derived; an R^2^ value of “–” indicates that the data in that group was unable to be fit to any model of that type that would perform better than a horizontal line set at a constant value, such as the mean of the data group.

**Table 1:** Number of exposures and respective R^2^ values for all 2D models with power and sigmoidal functions in front and side orientation groups.

Plot type	Number of exposures	R^2^
Power function, side orientation	1	0.440
	2	0.508
	3	0.679
	4	0.496
	14	0.223
	30	N/A
	All	0.479
Power function, front orientation	1	0.552
	2	0.478
	3	0.423
	4	0.333
	All	0.283
Sigmoidal function, side orientation	1	0.402
	2	–
	3	–
	4	–
	14	–
	30	–
	All	–
Sigmoidal function, front orientation	1	–
	2	0.264
	3	–
	4	–
	All	–

**Table 2:** Number of exposures and respective Area under the curve for ROC curves based on 3D models with power and sigmoidal functions in front and side orientation groups.

Model type	Interaction term	Orientation	Area under ROC curve
Power function	No interaction term	Front	0.727
		Side	> 0.999
	With interaction term	Front	0.873
		Side	> 0.999
Sigmoidal function	No interaction term	Front	0.500
		Side	0.500
	With interaction term	Front	0.500
		Side	0.500

As can be seen in Table 1, the power function models fit the various data groups far better than sigmoidal functions, most of which have “–”R^2^ values indicating they are worse fits than a constant function model. This result is also seen in Table 2, where the reported area under the ROC curves of the 3D models indicate that the power function models are much better at predicting whether or not lung injury is present than the sigmoidal models.

**Figure 5:**
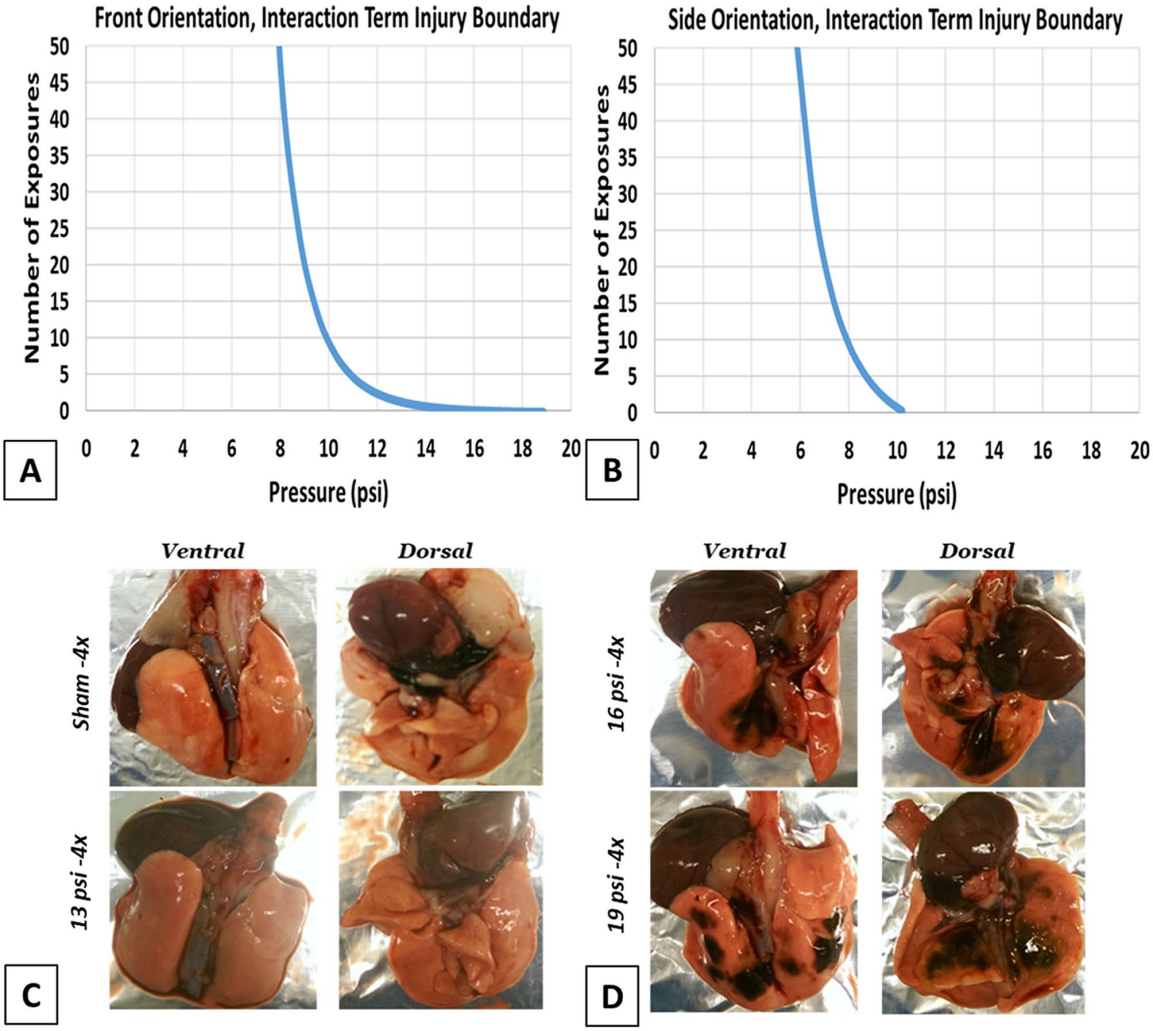
Boundary curves between not injured and injured based on number of exposures and pressure of those exposures for power curve 3D models with interaction terms. (**A**) front orientation model; (**B**) side orientation model. (**C** and **D**) Representative lung images of sham, 13, 16 and 19 psi from 4x blast group.

From the predictions of not injured/injured from the 3D power function models, an equation to define a boundary curve between not injured/injured based on pressure of exposures and number of exposures to that pressure was derived. These boundary curve plots are below in Fig. 5.

As can be seen in Fig. 5 and Table 2 above, for both the front and side orientations the models that include terms that capture an interaction effect between the number of exposures and the pressure of those exposures (in this model, roughly the same between exposures within a pressure group) provided a finer distinction between the combinations where injury to the lung does and does not occur. In the models with interaction terms, there is an approximate boundary of about 8 psi for multiple exposures when exposed from the front, while side orientations can have multiple exposures of approximately no more than 6 psi before damage occurs.

## Discussion

Single exposures to the low level BOPs experienced during training with breaching, firing of heavy weapon systems, or application of explosive devices such as grenades typically do not produce injury such as lung hemorrhage. However, since these training situations typically involve repeated BOP exposures, it is critical to consider potential cumulative deleterious effects, which have largely been overlooked in the experimental literature. The primary objective of this study was to define cumulative effects of repetitive BOP exposure on the lung and on this basis define the injury thresholds (i.e. non-auditory). While Bowen’s curves were originally developed for survivability, they were subsequently expanded to include lung injury, and are often considered as the “gold standard” of injury risk curves for acute BOP. There are limitations of the Bowen’s curves, as they were developed for a single exposure to a long duration blast overpressure, and have subsequently been curve-fitted with a limited data set to expand application to short duration BOP. To a large extent, this data is the basis for the lung injury standards described in Defense Explosives Safety Regulation (DESR) 6055.09 Edition 1. (DESR 2019)^12^. In the limited number of previous studies addressing repeated exposures to BOP, Dodd et al. exposed animals up to 100 times with exposure rates ranging from 4–10 exposures per day and developed injury risk curves for laryngeal trauma primarily in the absence of pulmonary trauma^2^. Although this study indicated that the sheep larynx is very sensitive to BOP, it is important to note that larynx injury has not been observed in humans with the same level of exposure. Rather than recording exposure intensity versus resultant lung injury, MacFadden et al., 2012 defined injury thresholds for lung injury for single exposure based on normalized Work, which is a difficult parameter upon which to base guidelines for Warfighters in a training setting^14^. Moreover, the repeated exposure curves in MacFadden et al., 2012, were derived from the Dodd et al. data set, which as noted above has laryngeal injury, not lung injury, as the primary end point^2,13^. To the best of our knowledge, at present there are no published data sets that address lung injury resulting from repeated exposures to BOP based upon experimental data.

In the current study we found that a single exposure to a 19 psi shockwave causes noticeable lung injuries in both frontal and side orientations, which in both circumstances were exacerbated with increasing numbers of exposures. A similar trend was observed in rats exposed to 16 psi overpressures. As anticipated, the side orientation had significantly greater (~ 1.5–2 times) surface lung contusions and hemorrhage at both 16 and 19 psi overpressure intensities than were observed with frontal orientation to the same respective BOP magnitudes and repetitions. This is potentially due to the larger surface area and associated loading of the thorax that is directly exposed to BOP in the side orientation. Interestingly, similar orientation effects and cumulative effects of repeated exposures were not observed with 13 psi exposures, but at least half of the experimental subjects did slightly exceed the 1% lung injury threshold when analyzed through gross surface injury (Fig. 2). In contrast, Yelverton IS^14^ scores revealed a significant increase in lung injury in comparison to respective control groups with 2 ×–4 × exposures to 13 psi overpressures. However, the Yelverton IS scores failed to reveal either orientation effects or cumulative injury effects across 1 ×–4 × repeated BOP exposures (Fig. 2). The predictive injury risk curves using mathematical modeling have calculated the injury threshold when extrapolated up to 50 times exposure, and shown that the thresholds are 8 psi and 6.5 psi for front and side BOP exposure, respectively. In addition, the shape of the side exposure risk curves are far steeper than the front exposure, demonstrating a higher vulnerability of side exposure even at low level exposures, which has to be further supported empirically with additional studies in future.

When rats were exposed to a total of 14 daily 10 psi BOP exposures, the extent of lung injury exceeded the defined injury thresholds of 1% hemorrhaged surface area using our MATLAB 2015b imaging determination as well as a Yelverton IS score greater than 3. Conversely, the rats subjected to either 14 or 30 daily 8.5 psi BOP exposures were observed to have no surface lung contusion/hemorrhage along with Yelverton IS scores negative for injury (i.e. ≤ 2), thus effectively identifying the pulmonary injury threshold at 8.5 psi for up to a total of 30 exposures (one exposure per day). These independent methodologies to assess pulmonary injury were closely correlated, as illustrated in Fig. 2. Histiocytosis and inflammation was significantly increased after single and repeated exposures to 16 and 19 psi BOP, and single side exposure of 13 psi. No histiocytosis or inflammation was observed in repeated exposures to 8.5 and 10 psi overpressure (Fig. 3). It is possible the histiocytosis, specifically for 1 day time-point – 4 times could be from resorption of chronic hemorrhage by alveolar macrophages and also from localized inflammation in the cases where significant hemorrhage was not observed. Interestingly, only the rats undergoing repeated exposures to 19 psi overpressures showed significant decrements in open field exploratory behavior. Overall, this data indicates that surface lung contusion/Yelverton scores are sensitive indicators of adverse pulmonary effects of BOP at 24 h post exposure and provide useful parameters to employ as injury criteria to define the cumulative ill effects of repeated BOP exposures.

While defined military guidelines (MIL-STD-1474E) address the impact on hearing and auditory function of noise from weapon systems and other high acoustic environments, similar guidelines targeting potential health hazards affecting other organ systems are generally absent^15^. Consideration of exposures to BOP, including the cumulative effects of repeated BOP exposures, are critical to the health hazard assessments that define the occupational standards required to maintain Warfighter readiness. Insights from these assessments of the effects of low level BOP exposures can also provide valuable insights into the nature of these injuries to primary care physicians who might treat civilian victims of terrorist attacks or role 1 care medics assisting both military and civilians in areas of combat and military operations. Injury risk curves based upon empirical data which, with mathematical algorithms, can define the relationships among blast exposure number and intensity and outcome in a straightforward chart analogous to the USN93 dive tables used by US Navy, will provide a valuable tool to optimally maintain Warfighter health and readiness in training and operational settings.

One of the objectives of this study was to show that the standard sigmoidal curves, often seen to predict the presence and extent of an injury (e.g., Hubbard et al.^16^), would be insufficient when examining both the BOP intensities and the number of exposures to those pressures. It was found that, especially in the case of 3D models, often even a constant model was a better fit than sigmoidal models. This study also tested the effectiveness of power curve functions in modeling lung injury and found them to be a particularly good fit, especially when including a term that took into account the interaction effects of pressure and number of exposures. Finally, 3D models were shown to have better fit to the data when compared to the 2D models, as they were able to simultaneously take into account both the pressure and number of exposures when deriving the models. The method described here is a good first step in deriving models that will delineate between when an injury is and is not likely to occur, and how severe of an injury it is if it does occur, based entirely on aspects of the BOP exposures alone. Once an appropriate model is determined, this method could be applied to multiple other biological systems after exposure, such as other internal organs and possibly even blood-based and other biomarker levels. This method has also determined the ranges of pressure and number of exposures in which lung polytrauma is less likely to be seen, allowing for a focus on testing criteria when examining the effects of low-level BOP on the brain. Applying this method further will help determine similar ranges for other biological systems. It was found that more exposures at a lower pressure will eventually result in pulmonary injuries, just as less BOPs at higher pressures would, as seen in Stuhmiller, 1996^17^. In the interaction term models found here, as the pressure of the exposures increased, percent lung injury greatly increased; as both the exposure pressure and the number of exposures increased, the percent lung injury increased even more rapidly.

The method of fitting the data to a curve in MATLAB 2015b is restricted to the ranges of the data itself. Testing of the models outside the data range (extrapolation) has been examined, and while the results of the models do hold for up to 50 exposures and 50 psi (that is, the predicted percent injury to the lungs would be expected for the pressure profile), outside of that range, no testing of the models has been performed yet. While the overall range the models were fit to extended from 0 to 22.5 psi and 1 to 30 exposures, the actual data to fit was confined to only 21 combinations in the side orientation and 16 combinations in the front orientation. In addition, the current exposure conditions are spaced 24 h apart, which in future studies would be altered to define outcome measures that can help Warfighter return-to-duty guidelines.

## Conclusion

In conclusion, we found with empirical data that the unscaled threshold for the repeated primary BOP exposure is 8.5 up to 30 BOP exposures. Modeling the data demonstrated that this may predict injury risk up to 50 exposures with 8psi predicted to to be the injury threshold for front exposures and 6psi to be the threshold for side exposures, which needs to be further validated with experimental data. Additional validation is also required for overpressure exposures associated with weapon systems (e.g. Carl Gustaf) that generate complex blast wave forms rather than the Friedlander waveforms considered in this study.

## Methods

All animal experiments were conducted under an approved animal use protocol in an AAALACi accredited facility in compliance with the Animal Welfare Act and other federal statutes and regulations relating to animals and experiments involving animals and adheres to principles stated in the Guide for the Care and Use of Laboratory Animals, NRC Publication, 2011 edition. Male Sprague Dawley rats, 8–9 weeks old that weighed ~ 275 g (Charles River Laboratories, Wilmington, MA), were housed two animals per cage at 20–22 °C (12 h light/dark cycle) with access to food (standard rat chow) and water ad libitum with bedding.

### Blast overpressure

Animals were anesthetized with isoflurane inside a closed Plexiglas chamber connected to a surgical vaporizer and subjected to survivable BOP using an ABS located at the Walter Reed Army Institute of Research (WRAIR). The ABS consists of a 0.5 ft long compression chamber that is separated from a 21 ft long transition/expansion test section by rupturable VALMEX membranes for pressures 13 psi or higher (Mehler texnologies, VA), or acetate membranes for pressures at 8.5 and 10 psi (Grafix Inc, OH). The anesthetized rat was secured in a longitudinal (i.e. head-on) or transverse (i.e. side-on) orientation in the middle of test section, as illustrated in Sajja et al.^11^. The compression chamber was pressurized with room air, causing the membranes to rupture at a pressure that is dependent upon the thickness of the specific membrane sheet(s) separating the two chambers, yielding a supersonic blast wave that impacts the experimental subject in the test section. To yield a range of overpressure exposures in these experiments, we used Valmax or Acetate membranes yielding peak positive static pressures within the range of 8.5 to approximately 19 psi with a positive phase duration of 3-5msec. The critical biomechanical loading to the experimental subject was determined from both the static (Ps) and dynamic pressures (Pd) of the BOP wave, which are fully recorded by a combination of side-on and head-on piezoresistive pressure gauges (Endevco, Troy, IN) using an Astro-med tmx-18 acquisition system at a 800,000 Hz sampling rate^11,18^. Following 4% isoflurane anesthesia for 6 min, animals (n = 6 per group) were exposed to either a single exposure (1 ×), 2 exposures (2 ×; one daily exposure), 3 exposures (3 ×; one daily exposure), or 4 exposures (4 ×; one daily exposure) to assess the extent of gross lung injury as the primary outcome dependent measure^19^. To determine the lung injury risk curves for repeated exposures in small animal model, animals were exposed to either 14 total exposures (14 ×; one daily exposure) or 30 total exposures (30 ×; one daily exposure) with breaks over the weekends (Table 3). All sham animals in these experiments are subjected to isoflurane anesthesia, loading in the shock tube, and recovery procedures as described above, but are not exposed to BOP. The defined end-point of this work is to identify at least 1% lung injury threshold for any given exposure condition up to 30 ×. Although the end-points have been achieved with single exposure above 13 psi, additional research with repeated exposures up to 4 times was conducted at pressure above 13 psi to have sufficient data points to mathematically model the data.

**Table 3:** Distribution of rodents into the various pressure, number of exposures, and orientation combinations.

	Pressure (psi)	0	8.5		10		12.5		16		19	
	Orientation	Sham	Front	Side	Front	Side	Front	Side	Front	Side	Front	Side
Number of exposures	1	6	–	–	–	–	6	6	6	6	6	6
	2	6	–	–	–	–	6	6	6	6	6	6
	3	6	–	–	–	–	6	6	6	6	6	6
	4	6	–	–	–	–	6	6	6	6	6	6
	14	6	–	6	–	6	–	–	–	–	–	–
	30	6	–	6	–	–	–	–	–	–	–	–

### Behavioral assessments

#### Open field activity

Each experimental subject was individually placed in the rectangular Plexiglas (Omnitech Electronics, Inc, Columbus, Ohio) arena chamber instrumented with an array of infrared-based photobeams, which break up on movement/activity. The automated system via computer (Fusion Software, Omnitech Electronics, Inc, Columbus, Ohio, Link: https://www.omnitech-electronics.com/product/Fusion-Software/510) recorded photobeam breaks from the multiple animals’ movement across multiple arenas simultaneously for 5 min at 24 h following the final BOP exposure.

### Euthanasia and tissue collection

Animals were euthanized by inhalation of 5% isoflurane for at least 8 min inside a closed Plexiglas chamber connected to a surgical vaporizer. Following the cessation of breathing, and in the absence of toe pinch reflex, thoracotomies were performed and lungs were exposed and photographed to assess gross damage; lungs were insufflated with 4% paraformaldehyde to fix the tissue for further evaluation.

### Macroscopic lung assessments

During photography of the lung tissue, a ruler was utilized to scale assessments of the gross hemorrhage and contusions on the dorsal and ventral surfaces of each animal’s lungs. Extent of injury was calculated from custom code of MATLAB 2015b (link: https://www.mathworks.com/help/matlab/release-notes-R2015b.html) with the image processing toolbox. Percentage of injury = (area of injury/total surface area) × 100. Lung injury was also scored using the method developed by Yelverton for blast lung injuries (Yelverton et al. 1996). The injury severity (IS) was defined by the equation: IS = (E + G + ST) × (SD), where E is the extent of injury to the lungs (range of 0–5), G is the injury grade, including the surface area of the lesions (range of 0–4), ST is the severity type, and SD is the severity depth element, indicating the depth or the degree of disruption of the worst case lesion (range of 1–4). The Yelverton lung injury score is defined as negative (IS = 0–2), trace (IS = 3–4), slight (IS = 5–21), moderate (IS = 22–36), and extensive (IS ≥ 37), depending on the severity of the injury.

### Pathological assessment

Hematoxylin and eosin (H&E) stained sections were evaluated by a board certified veterinary pathologist, blinded to animal treatment, at WRAIR to assess for inflammation and histiocytosis, using the following grading scheme: 0—absent; 1—minimal (< 10% of tissue section affected); 2—mild (11–25% of tissue section affected); 3—moderate (26–50% of tissue section affected); 4—marked (51–74% affected); 5—severe (> 75% of tissue section affected).

### Mathematical modeling

The data were sorted into 33 groups based on each subject’s orientation, number of exposures, and exposure pressures, and the averages for the lung injury data and pressure were calculated for each group. The average data were then imported into MATLAB 2015b (Mathworks, Natick, MA), along with the full data set. Both data sets were separated in MATLAB 2015b into individual independent and dependent variables. Next the two- and three-dimensional sigmoidal and power models were defined, both without and with interaction terms, for later use in the MATLAB 2015b methods; these models were:

- 2D:
  - Sigmoidal
    1 $$f\left(x\right)=\frac{1}{1+{e}^{-ax}}+b$$
  - Power
    2 $$f\left(x\right)=a{x}^{b}+c$$
- 3D:
  - Sigmoidal
    3 $$f\left(x, y\right)=\frac{1}{1+{e}^{-ax}+{e}^{-by}}+c$$
    4 $$f\left(x, y\right)=\frac{1}{1+{e}^{-ax}+{e}^{-by}+{e}^{-cxy}}+d$$
  - Power
    5 $$f\left(x,y\right)=a+b{x}^{c}+d{y}^{k}$$
    6 $$f\left(x,y\right)=a+b{x}^{c}+d{y}^{k}+m{x}^{p}{y}^{q}$$

In the models, *x* is the pressure of the simulated BOP wave(s), *y* is the number of BOP exposures at that pressure, *f*(*x*) and *f*(*x*, *y*) are the estimates of lung injury (which are then converted to percentage), and *a*, *b*, *c*, *d*, *k*, *m*, *p*, and *q* are coefficients for the model computed in MATLAB 2015b. In the 3D models, Eqs. (4 and 6) incorporate a term for describing any interactions between the pressure of the exposures and the number of exposures to that pressure. Using MATLAB 2015b’s curve fitting toolbox, the average data set was fit to each of these models for each orientation separately (sham data was used with both orientations sets); this was due to the large difference seen between the orientations in the lung contusion data, when pressure group and number of exposures was held constant. For the 2D models, fitting was done for the groups defined by number of exposures separately, resulting in models defined by 1 exposure, 2 exposures, etc., as well as for all data for an orientation, regardless of number of exposures; this was done to examine the effect of number of exposures on the variables. For the 3D models, all values for an orientation were used at once. Once models were found for the average data sets, the coefficients from these models were stored; the full data sets for each variable were then fit to the above models, using the coefficients from the average data sets as the coefficient initial value estimates in the iterative methods defined using MATLAB 2015b toolboxes.

The R^2^ values for the 2D models, used as an indicator of how well the models fit, were calculated and saved. The equations defining the best-fit 3D models were used to interpolate (and in the case of the front orientation, extrapolate as well) values for the percent lung contusion for number of exposures ranging from 1 to 30 and pressures from 0 to 20 psi in increments of about 0.75 psi for both front and side orientations, and for models with and without interaction terms. The estimated values, as well as the actual values, were determined to be pass/fail based on known values (here, any lung damage was a fail), and receiver operating characteristic (ROC) curves, as well as the areas under these curves (AUCs), were computed to determine the quality of each 3D model. The models derived from each orientation were tested against combinations of both front and side orientation data, to prevent the models from being tested against the same data used to create those models (which would have resulted in near-perfect accuracy). These pass/fail predications were also used to define a boundary curve based on exposure pressure and number of exposures, where one side of the curve shows combinations of number of exposures and pressure where failure/injury is likely to occur; these curves were defined on the data points (pressure and number of exposures) where the transitions from not injured to injured occurred, and computed using MATLAB 2015b toolboxes.

### Data analysis and statistical evaluation

All the changes were assessed using one-way analysis of variance (ANOVA) with a Tukey post-hoc test using GraphPad Prism 7 software (https://www.graphpad.com/guides/prism/7/user-guide/index.htm). Yelverton scores were analyzed with the non-parametric Kruskal–Wallis test and it was determined that there were significant differences among groups (p < 0.001). Therefore, the non-parametric Mann–Whitney U test was performed to compare values of experimental groups versus shams. Adjusted p values for actual comparisons of interest were assessed and corrections for the several comparisons were performed where appropriate. Correlation was assessed between Yelverton score and MATLAB 2015b-based surface lung injury calculation using Pearson’s correlation and area under curve (AUC) of receiver operating characteristic (ROC) curve was assessed using GraphPad Prism 7 software. A significance level of p < 0.05 was considered statistically significant. Unless otherwise specified, all data are expressed as mean ± SEM.

### Disclaimer

Material has been reviewed by the Walter Reed Army Institute of Research. There is no objection to its presentation and/or publication. The opinions or assertions contained herein are the private views of the author, and are not to be construed as official, or as reflecting true views of the Department of the Army, Department of the Navy, or the Department of Defense. The views expressed in this manuscript are those of the author and do not necessarily reflect the official policy or position of the Department of the Navy, Department of Defense, nor the U.S. Government. The study protocol was reviewed and approved by the Walter Reed Army Institute of Research Institutional Animal Care and Use Committee in compliance with all applicable federal regulations governing the protection of animals and research.” Research was conducted under an approved animal use protocol in an AAALACi accredited facility in compliance with the Animal Welfare Act and other federal statutes and regulations relating to animals and experiments involving animals and adheres to principles stated in the Guide for the Care and Use of Laboratory Animals, NRC Publication, 2011 edition.. JL and SA are employees of the U.S. Government. This work was prepared as part of their official duties. Title 17, U.S.C., §105 provides that copyright protection under this title is not available for any work of the U.S. Government. Title 17, U.S.C., §101 defines a U.S. Government work as a work prepared by an employee of the U.S. Government as part of that person’s official duties.
